# Energy dissipation dataset for reversible logic gates in quantum dot-cellular automata

**DOI:** 10.1016/j.dib.2016.12.050

**Published:** 2016-12-29

**Authors:** Ali Newaz Bahar, Mohammad Maksudur Rahman, Nur Mohammad Nahid, Md. Kamrul Hassan

**Affiliations:** a Department of Information and Communication Technology, Mawlana Bhashani Science and Technology University, Bangladesh; b University Grants Commission of Bangladesh, Bangladesh

**Keywords:** Quantum-dot cellular, Reversible logic gate, QCAPro

## Abstract

This paper presents an energy dissipation dataset of different reversible logic gates in quantum-dot cellular automata. The proposed circuits have been designed and verified using QCADesigner simulator. Besides, the energy dissipation has been calculated under three different tunneling energy level at temperature *T*=2 K. For estimating the energy dissipation of proposed gates; QCAPro tool has been employed.

**Specifications Table**

**Table:** 

Subject area	*Electronics*
More specific subject area	*Nano-electronics*
Type of data	*Table, figure*
How data was acquired	*QCADesigner and QCAPro tools have been used to acquire the data set*
Data format	*Analyzed*
Data accessibility	*Data is within this article*

**Value of the data**

• This data can help researchers who are going to design ultra-low power reversible system.
• The proposed circuit layout can be employed to design efficient large scale reversible system.
• Nano-communication system can be easily designed by implementing the proposed reversible gates.

## 1 Data

This article describes the QCA implementation of the basic reversible gate such as: Double Feynman, Toffoli, TR, BJN, R, NG, SCL and BVF gates. Table 1 describe the energy dissipation dataset at different tunneling energy level, $\gamma =0.5E_{k},\gamma =1E_{k}$ and $\gamma =1.5E_{k}$.

## 2 Experimental design, materials and methods

### 2.1 QCA implementation

**Fig. 1:**
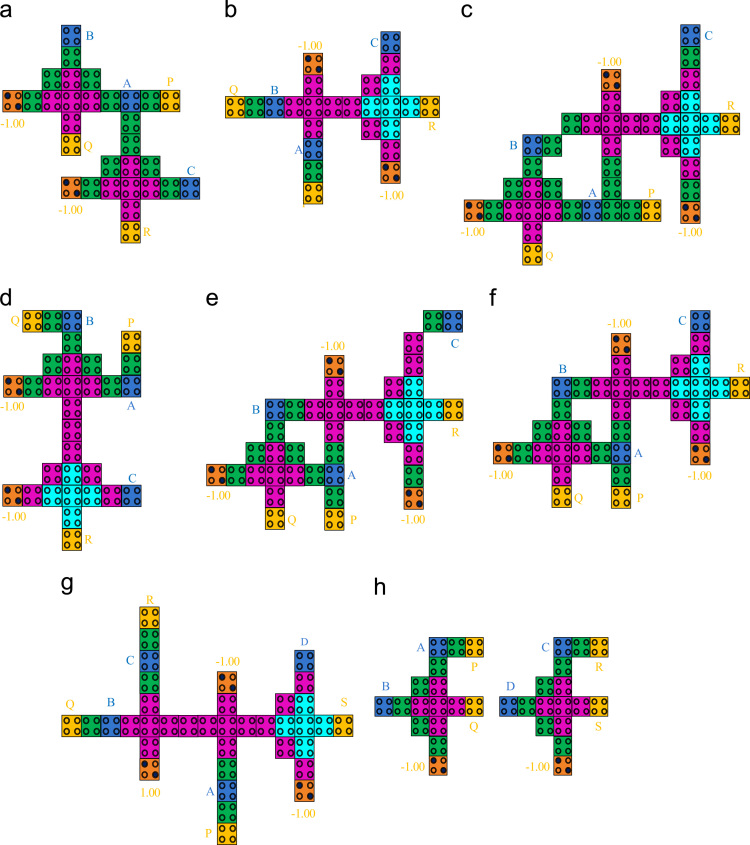
QCA circuit layout of proposed (a) Double Feynman gate (b)Toffoli gate (c)TR gate (d)BJN gate €R gate (f)NG gate (g)SCL gate (h)BVF gate.

To design the proposed gates, a 5-input majority voter gate [1] based 3-input exclusive-OR gate has been used. QCADesigner with default simulation engine has been employed to simulate the proposed circuit layouts. The QCA representation of the proposed gates is shown in Fig. 1.

### 2.2 Power dissipation analysis

In order to estimate the energy dissipation of proposed circuits, QCAPro [2] a power analyzing tools has been employed. The energy dissipation is analyzed in three different tunneling energy levels at 2 K temperature. The power dissipation by a QCA cell is calculated using the Hartree-Fock approximation. The Hamiltonian matrix of a mean-field approach is illustrated as [2], [3], [4].

(1) $$H=\left[\begin{array}{cc} \frac{{-E}_{k}}{2}\sum_{i}C_{i}{\;f}_{i,\;j} & -\gamma \\ -\gamma & \frac{E_{k}}{2}\sum_{i}C_{i}{\;f}_{i,\;j} \end{array}\right]=\left[\begin{array}{cc} \frac{{-E}_{k}}{2}\left(C_{j-1}+C_{j+1}\right) & -\gamma \\ -\gamma & \frac{E_{k}}{2}\left(C_{j-1}+C_{j+1}\right) \end{array}\right]$$

According to the upper bound power dissipation model [2] the power dissipation by a QCA cell is given as

(2) $$P_{\mathrm{diss}}=\frac{E_{\mathrm{diss}}}{T_{\mathrm{cc}}}\langle \frac{\hbar }{{2T}_{\mathrm{cc}}}{\vec{Г}}_{\;+}\times \left[-\frac{{\vec{Г}}_{\;+}}{\left|{\vec{Г}}_{\;+}\right|}\tan h\left(\frac{\hbar \left|{\vec{Г}}_{\;+}\right|}{k_{B}T}\right)+\frac{{\vec{Г}}_{\;-}}{\left|{\vec{Г}}_{\;-}\right|}\tan h\left(\frac{\hbar \left|{\vec{Г}}_{\;-}\right|}{k_{B}T}\right)\right]\rangle$$

## Figures and Tables

**Table 1:** Energy dissipation analysis of proposed reversible logic gates at three different tunneling energy levels.

Circuit	Leakage energy dissipation (meV)			Switching energy dissipation (meV)			Total energy dissipation (meV)		
	0.5 *E*_*k*_	1.0 *E*_*k*_	1.5 *E*_*k*_	0.5 *E*_*k*_	1.0 *E*_*k*_	1.5 *E*_*k*_	0.5 *E*_*k*_	1.0 *E*_*k*_	1.5 *E*_*k*_
Double Feynman gate	3.49	9.71	16.54	21.78	19.43	16.29	25.27	29.14	32.83
Toffoli gate	2.65	7.58	13.44	16.79	14.92	12.85	19.44	22.5	26.29
TR gate	5.06	15.95	27.20	34.80	30.66	26.38	39.87	46.62	53.59
BJN gate	3.23	8.97	16.04	19.65	17.59	15.18	22.88	26.56	31.22
R gate	4.98	14.68	26.95	33.82	29.74	25.39	38.8	44.42	52.34
NG gate	4.37	13.99	25.83	32.21	27.98	24.29	36.58	41.97	50.12
SCL gate	2.97	8.91	15.17	18.29	17.19	15.88	21.26	26.1	31.05
BVF gate	3.08	9.7	16.54	21.16	18.64	16.04	24.24	28.34	32.58
