# Supplementary material for: Energy dissipation dataset for reversible logic gates in quantum dot-cellular automata
Source: Data Brief. 2016 Dec 29;10:557–60. doi: 10.1016/j.dib.2016.12.050 (PMC5219615; doi:10.1016/j.dib.2016.12.050)

Conflict of Interest

We wish to confirm that there are no known conflicts of interest associated with this publication and there has been no significant financial support for this work that could have influenced its outcome.

We confirm that the manuscript has been read and approved by Ali Newaz Bahar, Mohammad Maksudur Rahman, Nur Mohammad Nahid and Md. Kamrul Hassan and that there are no other persons who satisfied the criteria for authorship but are not listed. We further confirm that the order of authors listed in the manuscript has been approved by all of us.

We confirm that we have given due consideration to the protection of intellectual property associated with this work and that there are no impediments to publication, including the timing of publication, with respect to intellectual property. In so doing we confirm that we have followed the regulations of our institutions concerning intellectual property.

We understand that the Corresponding Author is the sole contact for the Editorial process. He is responsible for communicating with the other authors about progress, submissions of revisions and final approval of proofs. We confirm that we have provided a current, correct email address which is accessible by the Corresponding Author and which has been configured to accept email from bahar_mitdu@yahoo.com.

Signature of Authors:

Ali Newaz Bahar

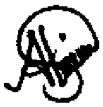

Mohammad Maksudur Rahman

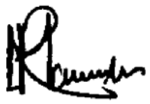

Nur Mohammad Nahid

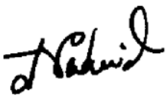

Md. Kamrul Hassan

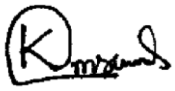

Supplement: Supplementary file 1 — Supplementary material [file mmc1.pdf]
